# Proprioceptive and Clinical Outcomes after Remnant Preserved Anterior Cruciate Ligament Reconstruction: Assessment with Minimal Confounding Factors

**DOI:** 10.1111/os.12763

**Published:** 2021-12-04

**Authors:** Yufeng Liu, Chunbao Li, Ning Ma, Wei Qi, Feng Gao, Bo Hu, Baiqing Zhang, Zhongli Li, Yujie Liu, Min Wei

**Affiliations:** ^1^ Department of Sports Medicine Chinese PLA General Hospital Beijing China; ^2^ Department of Sports Medicine and Rehabilitation Institute of Orthopedic, Chinese PLA General Hospital Beijing China; ^3^ Department of Sports Injury and Arthroscopy Surgery National Institute of Sports Medicine Beijing China; ^4^ The Second Department of Orthopedics Beijing Chaoyang Integrative Medicine Emergency Medical Center Beijing China

**Keywords:** Anterior Cruciate ligament, Anterior cruciate ligament reconstruction, Proprioception, Remnant preservation

## Abstract

**Objective:**

To evaluate the proprioceptive and clinical function of the knee joint after anterior cruciate ligament reconstruction (ACLR) with various amounts of remnant preserved with as few confounding factors as possible.

**Methods:**

This retrospective study included 46 patients who underwent ACLR with remnant preservation between March 2013 and February 2019. These patients had less than 6 months injury‐to‐surgery interval and no concomitant injuries. The researchers divided these subjects into two groups based on the length of the remnant preserved after ACLR, with group A defined as having more than 1/3 of the original length preserved and group B defined as less than 1/3 of the original length preserved. Clinical scores were obtained using the Lysholm knee scoring scale and the Tegner activity scale. The Lysholm score was calculated preoperatively, at 3, 6, and 12 months postoperatively, and at the last follow up. The Tegner score was calculated preoperatively, at 12 months postoperatively and at the last follow up. Anterior laxity was measured using the KT2000 arthrometer preoperatively and at 12 months postoperatively. Proprioceptive function was evaluated through reproduction of passive positioning (RPP) and threshold to detection of passive motion (TDPM). Both RPP and TDPM were measured at the angle of 15° at 3, 6, and 12 months postoperatively. Unpaired *t*‐tests were performed to investigate the difference in each parameters between the two groups.

**Results:**

In the present study, 20 patients were classified into group A and 26 into group B. All patients were followed up for an average of 34.70 ± 12.79 months. All 46 patients were satisfied with the outcome of the surgery and no complications were reported at the end of the study. No significant differences were found between the two groups in terms of the Lysholm score and anterior laxity by KT2000 at all time points. The Tegner score was significantly higher in group A at 12 months postoperatively and at the final follow‐up. In addition, group A's RPP was significantly better than that of group B's when tested at the angles of 15° and 30° at 3 months postoperatively, and at the angle of 15° at 6 months postoperatively. Group A's TDPM was also significantly better than that of group B's at all three tested angles at 3 months postoperatively, and at the angle of 15° at 6 months postoperatively.

**Conclusion:**

Patients with ACLR with more than 1/3 of the original length preserved demonstrated a higher activity level 12 months postoperatively and better proprioceptive function at 15° of extension at 3 and 6 months postoperatively.

## Introduction

The anterior cruciate ligament (ACL) is the major mechanism that maintains the stability of the knee joint. As one of the most common sports‐related injuries, ACL ruptures can result in laxity of the knee joint. Surgical reconstruction of the ACL is a common procedure that rebuilds the ACL with various types of grafts, aiming to reestablish the static stability of the knee joint to avoid further risks and additional damage. However, the inability for many to return to pre‐injury levels of sport largely undermines the efficacy of this procedure for professional athletes and patients with a high demand for sports. In addition, a higher rate of re‐tear after ACL reconstruction (ACLR) also calls for improvements in the current treatment approach. As well as the restoration of mechanical stability, the recovery of proprioception of the joint, which is frequently neglected but plays a big part in the function of the knee joint, is also considered a critical and requisite goal of this procedure. Proprioception is “the sense of position and movements of the limb”^1^ that is composed of three main modalities: awareness of the static position of the joint, the sense of movement and velocity of the joint (kinesthesia), and sensation of force^2^. It is the main mechanism that maintains the dynamic stability of the knee joint.

The ACL plays an important role in the proprioception of the knee joint^3^, as it contains a significant number of mechanoreceptors (MRC), including Ruffini nerve endings, Pacini receptors, and Golgi tendon organ‐like endings^4^, ^5^. MRC are the sensory receptors of proprioception and are essential for motor planning (feed forward for anticipation, preparation, and response planning) as well as rapid wiring into adaptation mechanisms to affect performance changes during task execution (feedback)^6^. ACL injuries can not only compromise the restraining force on the tibia but also damage and cause loss of MRC, causing consequential disturbance of the neuromuscular control of the affected knee^7^, which can lead to increased risk of reinjury of the affected knee and development of osteoarthritis.

Numerous methods of proprioceptive evaluation have been used, with a focus on specific parts of proprioception. Among these, joint position sense (JPS) and threshold to detection of passive motion (TDPM) are the most widely used approaches, and have established their credibility as the most representative indexes. JPS represents patients' ability to perceive the position of the joint without the help of other sensory mechanisms and only with the proprioception of the tested joint. The quantification of JPS is achieved through the measurement of the discrepancy of the reproduction of passive position (RPP), which is the difference between the pre‐set angle and the angle the patient attempts to reproduce through passive movement of the ipsilateral limp using certain equipment. TDPM is the ability to perceive the dynamic motion of the joint without the help of other sensory mechanisms and only the proprioception of the tested joint. The quantification of the TDPM is achieved through the measurement of the time or angle required for the patient to perceive the start of passive movement of the ipsilateral limp using certain equipment. However, there is a lack of uniform standards for other factors that might influence the outcome of these indexes, such as the tested angle of the joint, the direction or angular velocity of joint movement, and the position of the body, making the results of different studies less comparable.

Theoretically, the preservation of the remnant could contribute to the recovery of proprioception as the MRC in ACL were mostly found close to the bony insertion site of both the femur and the tibia^8^, ^9^, ^10^. In addition, remnant preservation has been reported to also enhance revascularization of the graft and prevent tunnel enlargement by reducing fluid leakage into the tunnels, which contribute to achieve better clinical outcomes. As a result, ACLR with remnant preservation has been drawing increasing attention. In a standard ACLR, the stump is removed to better expose the bony landmark of the tibial insertion and to avoid cyclops lesions. However, several studies demonstrate a deficiency of proprioception in standard ACL reconstructed knees with various types of grafts^11^, ^12^, ^13^, ^14^. In addition, aberrant gait biomechanics caused by somatosensory dysfunction following standard ACLR might lead to consequent osteoarthritis^15^. With better understanding of the anatomy of ACL insertion, and no evidence showing that remnant preservation could increase the incidence of cyclops lesions, remnant‐preserving ACLR is becoming increasingly popular. Many studies report the clinical outcomes of remnant‐preserving ACLR, but most focus on the clinical evaluation of mechanical stability, which is not always in accordance with the functional outcome and patients' satisfaction. The several studies that do assess proprioception recovery share a common flaw of loose controls for patient enrollment in terms of associated meniscus or cartilage injuries, as well as discrete injury‐to‐surgery time intervals; hence, too many confounding factors can sabotage the credibility of their conclusions.

The purpose of this study was to: (i) retrospectively analyze the clinical results of remnant‐preserving ACLR using the Lysholm score, the Tegner score, and anterior laxity by KT2000; (ii) evaluate the proprioceptive function of the knee joint after remnant‐preserving ACLR based on discrepancy of RPP and TDPM with as few confounding factors as possible; and (iii) explore the appropriate amount of remnant to be preserved. We hypothesized that the proprioceptive function of the knee joint showed a better result with more remnant preserved.

## Material and Methods

### Subject

**Fig 1:**
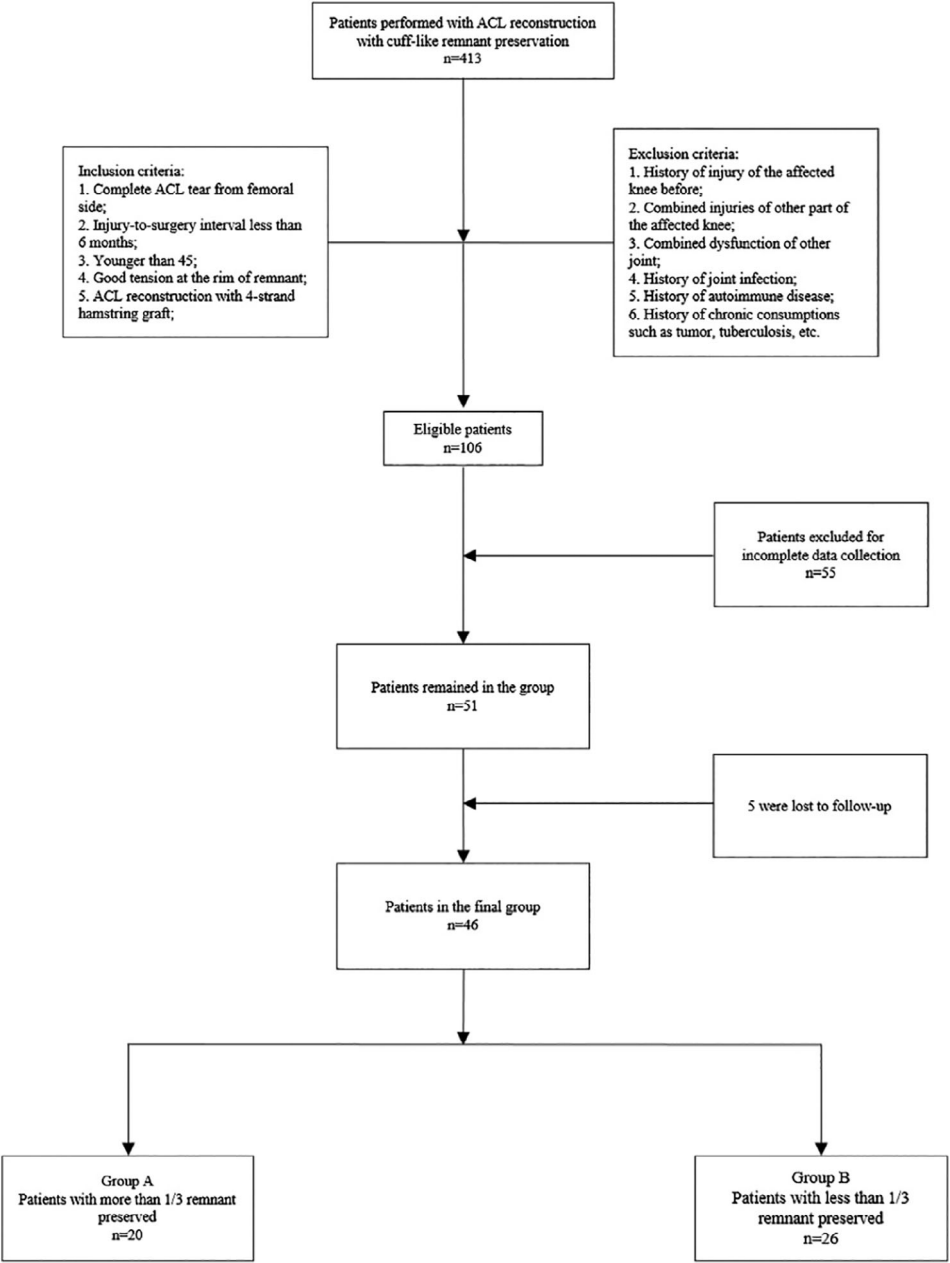
Flow chart of enrollment of patients.

This study was a retrospective study. All patients who underwent ACLR with cuff‐like remnant preservation from March 2013 to October 2019 were screened for inclusion in the study. The screening process is shown in Fig. 1.

### Inclusion and Exclusion Criteria

Inclusion criteria: (i) complete ACL tear from the femoral side; (ii) injury‐to‐surgery interval less than 6 months; (iii) younger than 45 years; (iv) good tension at the rim of the remnant; and (v) ACL reconstruction with four‐strand hamstring graft.

Exclusion criteria: (i) history of injury of the affected knee; (ii) combined injuries of other parts of the affected knee; (iii) combined dysfunction of other joints; (iv) history of joint infection; (v) history of autoimmune disease; and (vi) history of chronic wasting diseases, such as tumors and tuberculosis.

The injury‐to‐surgery interval was counted as 1 month if it's shorter than 1 months. All data were collected following approval by the institutional review board and ethics committee of the authors' institutions. All operations were performed by the same surgeon (Dr Min Wei, the corresponding author).

**Fig 2:**
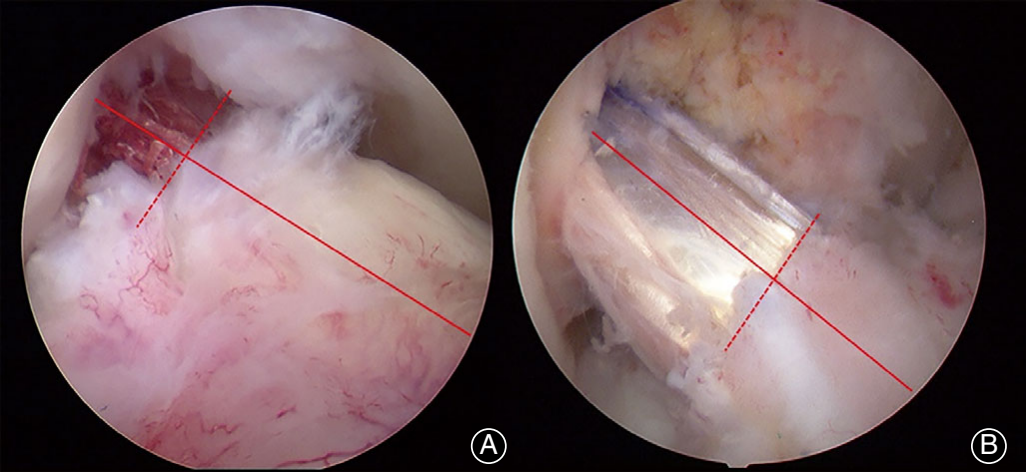
Arthroscopic image after anterior cruciate ligament reconstruction. (A) Reconstructed graft enveloped with more than 1/3 remnant preserved. (B) Reconstructed graft enveloped with less than 1/3 remnant preserved.

Depending on the length of the tibial remnant stump preserved, patients were divided into two groups: group >1/3, with more than 1/3 remnant preserved compared to the original length of the intact ACL; group <1/3, with less than 1/3 remnant preserved. Measurement of the length of the remnant was performed through arthroscopic measurement after reconstruction (Fig. 2).

### Surgical Technique

#### Anesthesia and Position

Under general anesthesia, the patient was placed in supine position, with the affected limb disinfected and properly draped, dangling at the edge of the table. The stability of the affected knee was examined under anesthesia.

#### Creation of Portals

The regular anteromedial (AM) and anterolateral (AL) portals were taken on the affected knee. The AL portal was created 1 cm lateral to the palpable patellar tendon 1 cm above the joint line. The AM portal was created in the same manner, only 1 cm medial to the palpable patellar tendon, which was also placed 1 cm above the joint line.

#### Surveillance of Intra‐articular Structure

**Fig 3:**
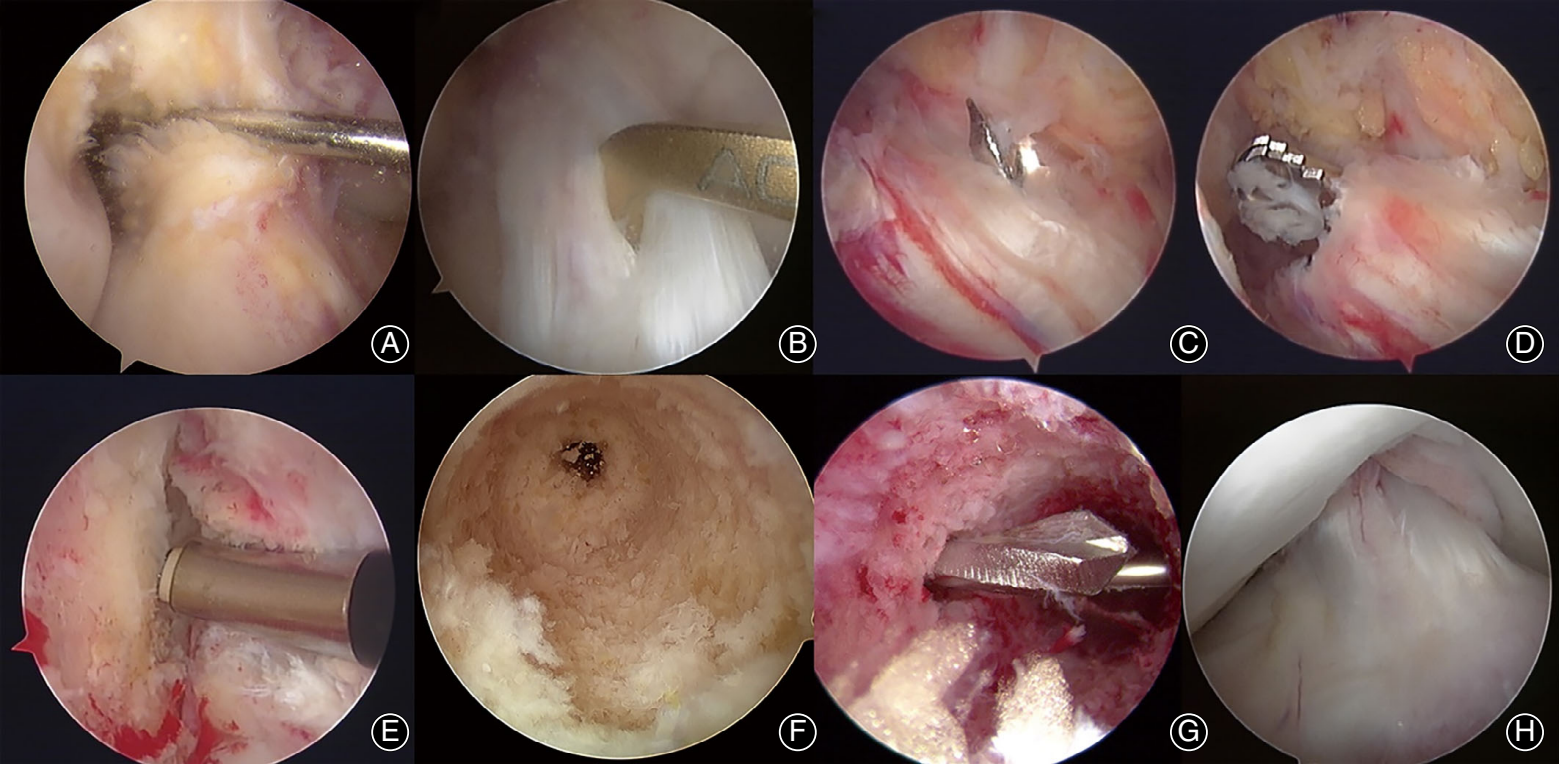
Arthroscopic images of the arthroscopic process of remnant‐preserved anterior cruciate ligament reconstruction (ACLR). (A) Confirmation of anterior cruciate ligament (ACL) rupture from the femoral insertion. (B) Pointing the tip of point‐to‐point guide for tibial tunnel into synovial sheath of the remnant stump at the center of footprint. (C) Penetration of the tibial cortex when drilling the tibial tunnel. (D) Clearing the inside of the remnant stump with a shaver. (E) Debridement of the femoral insertion. (F) Femoral tunnel completed. (G) Tunnel for the pin of RIGIDFix. (H) Impingement test after graft fixation.

Intra‐articular inspection was performed to rule out comorbidities, such as meniscus abnormality or chondromalacia. The site of the ACL injury was confirmed to be on the femoral side, leaving a relatively intact tibial side remnant (Fig. 3A). The condition of the remnant was evaluated to make sure the synovial sheath wrapping around the remnant remained intact.

#### Creation of Tibial Tunnels, Management of Remnant, and Creation of Femoral Tunnel

**Fig 4:**
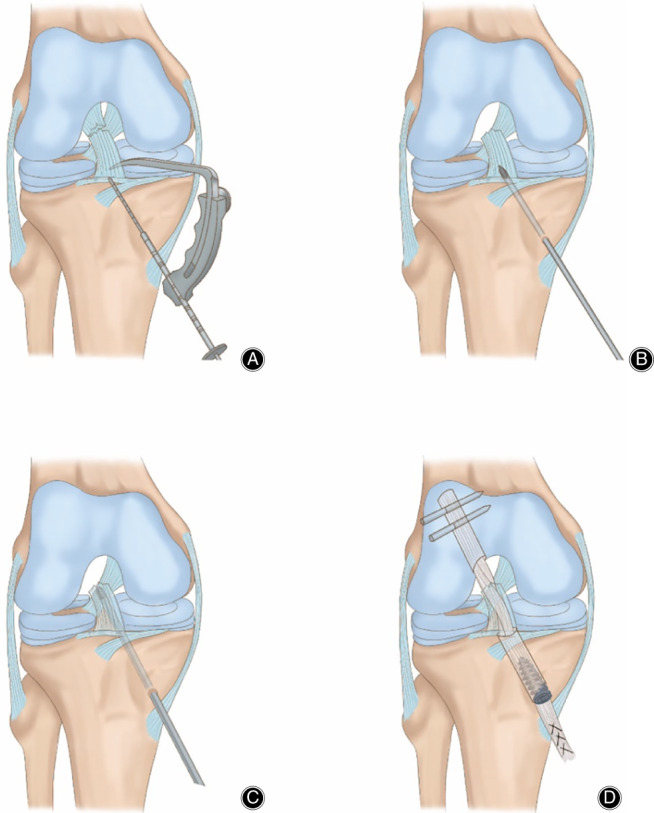
Diagram of the arthroscopic surgery. (A) Pointing the tip of point‐to‐point guide for tibial tunnel into synovial sheath of the remnant stump at the center of the footprint. (B) Penetration of the tibial cortex when drilling the tibial tunnel. (C) Clearing the inside of the remnant stump with a shaver. (D) Fixation of the graft.

Semitendinosus and gracilis tendons were harvested. The harvested tendon was braided into a four‐strand graft and tensioned, and left for later use. The tibial tunnel was created first. The intra‐articular position of the tibial tunnel was set slightly anterior to the center of the tibial footprint of the ACL. A probe was used to create a 2‐mm opening at the front of the synovial sheath of the remnant stump to place the tip of the point‐to‐point guide for tibial tunnel inside the footprint (Figs 3B and 4A). Drilling of the tibial tunnel stopped immediately after penetrating the tibial cortex (Figs 3C and 4B). The shaver was placed through the tibial tunnel to reach the center of the remnant. The inside of the remnant stump was cleared with a shaver (Figs 3D and 4C). Extra caution should be taken to preserve as much tissue and synovial sheath enveloping the stump as possible, especially the tension around the proximal opening of the sheath. The femoral tunnel was located at the center of the femoral footprint of the original ACL (Fig. 3E). The tunnel was drilled inside‐out (Fig. 3F).

#### Placement and Fixation of Graft

The braided graft was brought in through the distal entrance of the tibial tunnel, passing through the remnant stump. Due to the tension around the proximal opening of the synovial sheath of the stump, the remnant was pulled towards the femoral tunnel alongside the graft (Fig. 4D). After the graft was in place, the graft was fixed with RIGIDfix (MiTek, Depuy Synthes, Indiana, US) crosspin through the femoral tunnel (Fig. 3G and 4D). The knee was flexed to 30° and the graft was fixed with an interference screw (Smith Nephew, London, Britain) through the tibial tunnel (Fig. 4D). The tension of the graft was checked under arthroscope. No impingement was spotted with examination of flexion and extension of the knee (Fig. 3H). The Lachman test and the anterior drawer test were performed, and the results were negative. The distal entrance of the tibial tunnel was covered with the residual sartorius aponeurosis, which was sutured with the end of the graft.

#### Postoperative Management

Straight‐leg raising exercise started immediately after the surgery to prevent the atrophy of the quadriceps muscles. Touch‐down weight‐bearing was allowed on the second day after surgery, with the hinged brace locked In extension while walking and standing. Partial weight‐bearing could be gradually commenced as tolerated. Hinged brace of the knee joint was equipped to stabilize the knee for 8 weeks. In the first 2 weeks, the brace was locked while sleeping. Range of motion (ROM) exercise was allowed and advised to avoid stiffness of the knee with the brace unlocked while resting. Attention was focused on regaining full extension. The goal was to achieve 0°–90° ROM at the first 4 weeks. Crutches were necessary at this point for consideration of balance. Full ROM was allowed and advised in the 8th week. Full weight‐bearing started in the 9th week, with squatting and leg presses done to regain the strength of the quadriceps muscle. Crutches could be worn. Patients were to avoid running and ambulated bike riding until the strength of the quadriceps muscle was recovered. A functional brace could replace the hinged brace if necessary. Physical activity could gradually resume after the 12th week and patients could return to their former level of physical activity at 9 months after surgery. A functional brace was advised through the first year during sport activities.

### Clinical and Functional Evaluation

#### Lysholm Score

The Lysholm score is used to evaluate the outcomes of knee ligament surgery in patients. It includes eight items that measure pain (25 points), instability (25 points), locking (15 points), swelling (10 points), limp (5 points), stair climbing (10 points), squatting (5 points), and need for support (5 points). The total score may range from 0 to 100: 100 is the best possible outcome; 91 to 100 points is considered excellent; 84 to 90 is good; 65 to 83 is fair; and 64 or less is unsatisfactory. Measurement of the Lysholm score was done preoperatively, at 3, 6, and 12 months postoperatively, and at the final follow up. Despite being one of the most frequently used assessment tools for the results of ACL reconstruction, it does have the disadvantages of only measuring activities of daily living (ADL). Therefore, the Tegner activity score was also measured.

### Tegner Activity Score

The Tegner activity score is a one‐item score that grades activity based on work and sports activities on a scale of 0 to 10. Zero represents disability because of knee problems and 10 represents national‐level or international‐level soccer. The Tegner activity score was measured preoperatively, at 12 months postoperatively, and at the final follow‐up.

### Anterior Laxity by KT2000 Arthrometer

**Fig 5:**
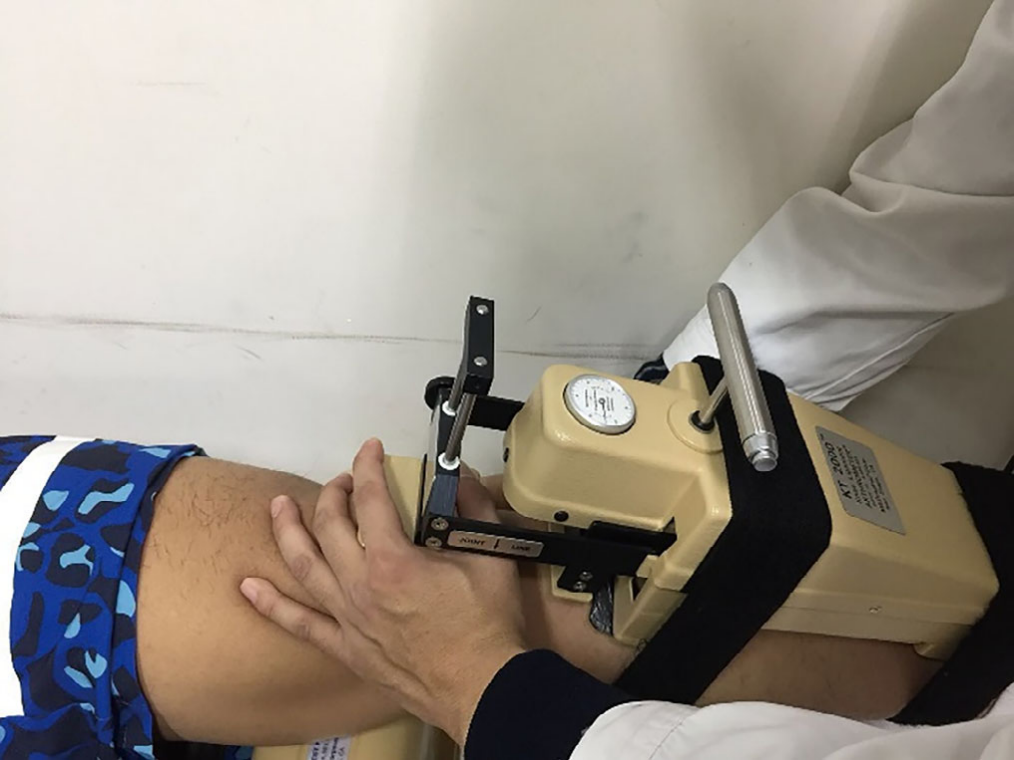
Measuring anterior laxity with KT2000 arthrometer (GENOUROB, Laval, France).

The KT2000 arthrometer (GENOUROB, Laval, France) was developed to provide objective measurement of the sagittal plane motions of the tibia relative to the femur. This motion, sometimes referred to as drawer motion, occurs when an examiner applies pulling force to the lower limb to move it forward. In this study, the KT2000 arthrometer was used to measure anterior laxity before surgery and at 12 months postoperatively (Fig. 5). The anteroposterior translation at 15lb (67N), 20lb (89N), and 30lb (135N) in 30° and 90° of flexion was measured, documented and statistically analyzed.

### Proprioceptive Evaluation

Proprioceptive evaluation included detection of static position and detection of dynamic motion at 3, 6, and 12 months after surgery, as it has been shown that reinnervation occurs approximately 6 months after ACLR^4^. The direction of both tests was set to be from 90° of flexion to full extension to avoid the confounding effect brought by the harvest of the hamstring.

### Discrepancy of Reproduction of Passive Position

**Fig 6:**
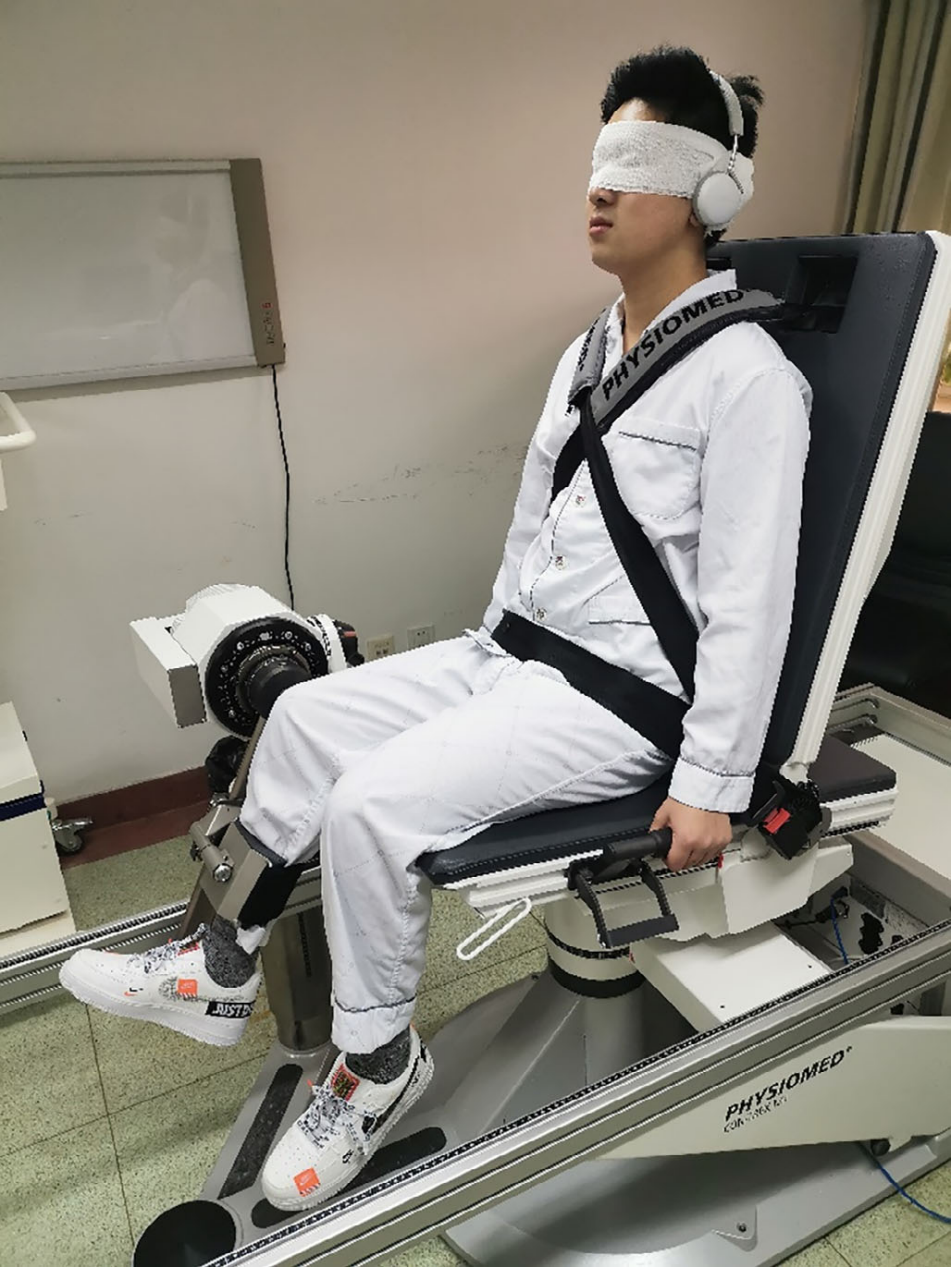
Measuring reproduction of passive positioning (RPP) and threshold to detection of passive motion (TDPM) using Con‐Trex MJ (Con‐Trex, Zürich, Switzerland). Patient was blindfolded and wearing headset with white noise to eliminate visual and auditory aids.

Detection of static position was evaluated through JPS, which was represented by discrepancy of RPP^16^. The method described by Barrack *et al*.^17^ using the isokinetic dynamometer Con‐Trex MJ (Con‐Trex, Zürich, Switzerland) was applied in this study (Fig. 6). The patient's visual and acoustical senses were blocked with bandages and headsets with white noise, respectively. The patient's affected knee was attached to the apparatus of the machine and placed at 90° of flexion at the beginning of the test. Angular velocity was set at 2°/s. The leg was passively extended to a certain angle by the apparatus and held for 5 s for the patients to memorize the joint position, then passively returned to the starting position. The session was repeated twice, and 2 min of rest followed to avoid confounding with neuromuscular fatigue. After that, patients were required to actively reproduce the target joint position five times, with 1‐min rest at each interval. The deviation between the reproduced and the original angle was measured. RPP at 15°, 30°, and 45° were tested and the mean value of the data produced five times was considered the final data.

### Threshold to Detection of Passive motion

Detection of dynamic motion was evaluated by TDPM. Lephart's method was utilized with the same machine used to measure JPS^18^ (Fig. 6). Patients were blindfolded, wearing a headset with white noise to block visual and acoustic affirmative. The affected leg was attached to the apparatus and passively set at a certain angle. Angular velocity was set at 0.25°/s from flexion to extension. The machine was started without any warning to the participants. Patients were asked to press the button once they detected the movement. The formal test was repeated five times, with 2 min of rest at interval. TDPM at 15°, 30°, and 45° were tested and the mean value of the data produced five times was used the final data.

### Statistical Analysis

Priori power analysis was performed using G*Power 3.1 (Heinrich Heine University Düsseldorf, German) to determine the sample size. The effect size was calculated according to the data in Lee's prior study^19^. For two‐tailed analysis with the power of 0.80, with α = 0.05, the estimated size of each group was 8 at minimum. Graphpad Prism 8.0 (Graphpad Software, California, USA) was used to analyze all the data collected. Normal distribution of all data was checked before subsequent analysis. The value of every affected factor was calculated and analyzed to determine the significance using Fisher's exact test and the unpaired *t*‐test with or without Welch's correction, with *P* < 0.05 considered statistically significant.

## Results

### Patient Demographics and Preoperative Parameters

From March 2013 to October 2019, 413 patients underwent ACLR with cuff‐like remnant preservation. A total of 106 patients were eligible for the study after screening according to inclusion and exclusion criteria. Among these, 55 were excluded because of incomplete data collection, and 5 were lost to follow up. Of the remaining 46 patients, 20 patients were defined as group >1/3, with more than 1/3 remnant preserved, and 26 as group <1/3, with less than 1/3 remnant preserved, which was far beyond the required size. All 46 patients were followed for an average of 34.70 ± 12.79 months. No complications were reported. Demographics and preoperative parameters are shown in Table 1. The age and injury‐to‐surgery interval of each group showed no statistical difference. The gender and affected side distribution in each group were not significantly different. Lysholm and Tegner scores showed no significant differences between each group. The maximum displacement of anterior laxity tested by KT2000 at 30° and 90° with the pulling strength at 15 lb (67 N), 20 lb (89 N), and 30 lb (135 N) were measured in each group, and no significant difference was found.

**TABLE 1:** Patient demographics^†^

	Group A (*n* = 20)	Group B (*n* = 26)	*P*‐value
Age (years)	29.8 ± 8.36 (16–44)	30.15 ± 8.63 (16–45)	0.8895^‡^
Male/female (*n*)	10/10	13/13	>0.9999^§^
Left/right side (*n*)	12/8	11/15	0.3726^§^
Months from injury (*n*)	3.56 ± 1.61	3.88 ± 1.68	0.4986^‡^
Follow‐up time	33.35 ± 13.13	35.73 ± 12.68	0.5373^‡^

^†^ Data were expressed as mean ± SD (range) unless otherwise indicated. SD, standard deviation.

^‡^ Unpaired *t*‐test. *P* < 0.05 was defined as significant.

^§^ Fisher's exact test.

### Postoperative Clinical and Functional Evaluation

The Lysholm score of each group revealed a significant increase between every time point. However, there was no statistically significant difference between the two groups (Table 2). Group >1/3 demonstrated significantly better Tegner score than group <1/3 at both postoperative time points (Table 3). With significantly decreased displacement at 12 months compared to the pre‐injury state, no statistical difference was found between the maximum displacement of anterior laxity tested by KT2000 for group >1/3 and group <1/3 (Table 4).

**TABLE 2:** Lysholm score at each time point^†^

	Group A (*n* = 20)	Group B (*n* = 26)	*P‐*value^‡^
Preoperation	38.55 ± 8.41 (25–53)	39.85 ± 7.8 (25–52)	0.592^ns^
3 months	79.85 ± 5.58 (71–88)	79.27 ± 5.49 (71–88)	0.7255^ns^
6 months	83.1 ± 5.34 (75–92)	83 ± 5.54 (75–93)	0.9511^ns^
12 months	88.5 ± 5.31 (82–98)	88.88 ± 5.49 (81–98)	0.8122^ns^
Last follow‐up	92.5 ± 5.32 (83–100)	91.3 ± 5.69 (82–100)	0.4725^ns^

^†^ Data were expressed as mean ± SD (range) unless otherwise indicated. SD, standard deviation.

^‡^ Unpaired *t*‐test. *P* < 0.05 was defined as significant.

ns, statistically non‐significant.

**TABLE 3:** Tegner score preoperation, 12 months postoperation, and at the last follow‐up^†^

	Group A	Group B	*P*‐value‡
Preoperation	1.55 ± 0.83	1.77 ± 0.82	0.3734^ns^
12 months	3.90 ± 1.45	5.04 ± 1.11	0.0042^**^
Last follow up	5.30 ± 1.26	6.39 ± 1.20	0.0048^**^

^†^ Data were expressed as mean ± SD (range) unless otherwise indicated. SD, standard deviation.

^‡^ Unpaired *t‐*test. *P* < 0.05 was defined as significant.

* *P* < 0.05.

** *P* < 0.01.

*** *P* < 0.001.

ns, statistically non‐significant.

**TABLE 4:** Anterior laxity by KT2000 preoperative and at 12 months postoperative ^†^

		Group A	Group B	*P*‐value
Preoperation	30° flexion			
	15 lb (67 N)	3.5 ± 1.64 (1–7)	3.31 ± 1.44 (0–6)	0.674^ns^
	20 lb (89 N)	4.40 ± 1.60 (2–7)	4.15 ± 1.54 (1–7)	0.6003^ns^
	30 lb (135 N)	5.4 ± 1.96 (3–11)	5.69 ± 1.76 (3–9)	0.5976^ns^
	90° flexion			
	15 lb (67 N)	2.55 ± 0.94 (1–4)	2.54 ± 1.10 (0–5)	0.9704^ns^
	20 lb (89 N)	3.65 ± 0.88 (2–5)	3.85 ± 1.32 (1–7)	0.5684^ns^
	30 lb (135 N)	4.3 ± 0.80 (3–6)	4.5 ± 1.24 (2–8)	0.5343^ns^
12 months	30° flexion			
	15 lb (67 N)	0.8 ± 0.77 (0–2)	1.08 ± 0.69 (0–2)	0.205^ns^
	20 lb (89 N)	1.5 ± 0.95 (0–3)	1.81 ± 0.90 (0–3)	0.2656^ns^
	30 lb (135 N)	1.95 ± 1.15 (0–4)	2.23 ± 1.11 (0–5)	0.4054^ns^
	90° flexion			
	15 lb (67 N)	0.45 ± 0.51 (0–1)	0.42 ± 0.58 (0–2)	0.87^ns^
	20 lb (89 N)	0.70 ± 0.57 (0–2)	0.65 ± 0.63 (0–2)	0.7986^ns^
	30 lb (135 N)	1.2 ± 0.77 (0–3)	1.12 ± 0.77 (0–3)	0.7123^ns^

^†^ Data were expressed as mean ± SD (range) unless otherwise indicated. SD, standard deviation.

^‡^ Unpaired *t*‐test. *P* < 0.05 was defined as significant.

ns, statistically non‐significant.

### Proprioceptive Evaluation

#### Discrepancy of Reproduction of Passive Positioning

At 3 months after surgery, group >1/3's RPP was significantly better than group <1/3's at the angles of 15° and 30°. At 45°, the RPP of group >1/3 demonstrated a higher accuracy than that of group <1/3 but without significant difference. At 6 months postoperatively, group >1/3's RPP was still significantly more accurate than group <1/3's when reproducing the angle of 15°. Group >1/3's RPP of 30° and 45° showed a better result without significant difference. At 12 months after surgery, even though group >1/3's RPP was more accurate than group <1/3's at all three tested angles, no significant difference was found (Table 5).

**TABLE 5:** Discrepancy of reproduction of passive positioning of 15°, 30°, and 45° at 3, 6, and 12 months postoperative^†^

Time of evaluation	Tested angle	Group	Mean angle discrepancy ± SD (°)	*P*‐value^‡^
3 months	15°	A	4.93 ± 0.92	0.0026^**^
		B	6.25 ± 1.82	
	30°	A	7.13 ± 1.04	0.0003^***^
		B	8.72 ± 1.71	
	45°	A	9.73 ± 1.44	0.6808^ns^
		B	9.93 ± 1.87	
6 months	15°	A	3.79 ± 1.14	0.0024^**^
		B	5.27 ± 1.94	
	30°	A	5.25 ± 0.96	0.4125^ns^
		B	5.60 ± 1.92	
	45°	A	7.03 ± 1.21	0.5187^ns^
		B	7.33 ± 1.91	
12 months	15°	A	3.44 ± 0.80	0.1236^ns^
		B	3.91 ± 1.25	
	30°	A	4.54 ± 1.05	0.1864^ns^
		B	5.02 ± 1.37	
	45°	A	5.56 ± 1.38	0.5461^ns^
		B	5.80 ± 1.33	

^†^ Data were expressed as mean ± SD (range) unless otherwise indicated. SD, standard deviation.

^‡^ Unpaired *t*‐test with Welch's correction.

^*^ *P* < 0.05.

** *P* < 0.01.

*** *P* < 0.001.

ns, statistically non‐significant.

### Threshold to Detection of Passive Motion

In regard to the TDPM, all three tested angles at 3 months showed significant superiority in group >1/3's data. At 6 months, the TDPM at 15° of group >1/3 was significantly smaller than that of group <1/3. Group >1/3's TDPM was smaller than that of group <1/3's without significant difference at 30° and 45°. After 12 months, no significant difference was found at all three tested angles, even though group >1/3's mean TDPM was smaller at any tested angle (Table 6).

**TABLE 6:** TDPM of 15°, 30°, and 45° at 3, 6, and 12 months postoperative^†^

Time of evaluation	Tested angle	Group	Mean TDPM ± SD (°)	*P*‐value^‡^
3 months	15°	A	2.03 ± 0.22	0.0010^**^
		B	2.42 ± 0.50	
	30°	A	2.22 ± 0.10	0.0002^***^
		B	2.66 ± 0.50	
	45°	A	2.56 ± 0.13	0.0001^***^
		B	2.94 ± 0.42	
6 months	15°	A	1.82 ± 0.10	0.0009^***^
		B	2.08 ± 0.36	
	30°	A	1.93 ± 0.09	0.2158^ns^
		B	1.99 ± 0.26	
	45°	A	2.05 ± 0.09	0.1334^ns^
		B	2.12 ± 0.22	
12 months	15°	A	1.63 ± 0.10	0.1511^ns^
		B	1.72 ± 0.29	
	30°	A	1.68 ± 0.12	0.1671^ns^
		B	1.77 ± 0.29	
	45°	A	1.80 ± 0.16	0.2403^ns^
		B	1.88 ± 0.31	

^†^ Data were expressed as mean ± SD (range) unless otherwise indicated. SD, standard deviation.

^‡^ Unpaired *t*‐test with Welch's correction.

^*^ *P* < 0.05

** *P* < 0.01

*** *P* < 0.001

**** *P* < 0.0001.

ns, statistically non‐significant; TDPM, threshold to detection of passive motion.

Proprioceptive evaluation was not performed at the last follow‐up, as less significant differences were found 12 months after surgery. It was reasonable to assume that significant differences after that, if ever, should be attributed to varying rehabilitation and individual variation rather than different amounts of remnant preserved.

## Discussion

The primary goal of this retrospective study was to evaluate the proprioception at different time points after ACLR with different amounts of remnant preserved with minimal confounding factors. No significant difference was found between the two groups' demographics preoperatively. More significantly, a superior proprioceptive outcome was found at the starting point (15°) of extension at 3 and 6 months after reconstruction with more than 1/3 remnant preserved. Significant difference between groups was found to be associated with the tested angle and time. In addition, the Tegner score was significantly higher in group >1/3 at 12 months postoperatively and at last follow up.

Even though a better proprioception recovery following ACLR with remnant preservation has already been demonstrated in several studies^19^, ^20^, ^21^, the appropriate amount of the remnant preserved remains controversial. Considering that the availability of the length of remnant varies among different injury patterns, it is unrealistic to apply a universal standard for various situations. However, the necessity of minimization of remnant debridement should be investigated. In the present study, group <1/3 demonstrated relatively inferior recovery of proprioceptive function, in terms of both JPS and TDPM. This finding is consistent with Lee's report^19^, ^21^. The significant differences of the present study are mostly concentrated at earlier time points for both RPP and TDPM. This phenomenon could be attributed to the amount of MRC residue in the remnant preserved. The regeneration of proprioception was obtained through the reinnervation of the graft after reconstruction. Ochi *et al*.^4^ found that it took 6 months for the graft to be reinnervated. Reider *et al*.^22^ also detected improvement in TDPM 6 months after reconstruction. One of the possibilities is that at 3 months after reconstruction, the graft was still not completely reinnervated and the proprioceptive function still largely relies on the residual MRC in the remnant. After the reinnervation was completed at 6 months, the MRC in the remnant contributed a relatively minor portion to the proprioceptive function, which is why the difference after 6 months was no longer significant. Another hypothesis is that a larger amount of remnant resulted in faster integration and reinnervation of the graft, increasing the proprioceptive function in the early phase of rehabilitation. It is reasonable to attribute this to a slower integration between the graft and remnant rather than a general inferior quality of integration based on a significantly bigger standard deviation of group <1/3. Gohil *et al*.^23^ found that the revascularization of the graft following minimal debridement of remnant was faster for serial MRI. In addition, a study on a sheep model demonstrated enhancement of cell proliferation, revascularization, and regeneration of proprioceptive organs in the reconstructed ACL with remnant preservation^24^. However, the preservation of remnant only helped to accelerate this process without increasing the amount of MRC in the reconstructed graft after the reinnervation was completed. As a result, no significant difference was found 12 months after surgery.

Another important finding was that JPS was more precise when more remnant was preserved at the starting point of ROM (15°). More significant results between groups were found concentrated at 15°. By contrast, less significant differences were found at 30°, and data of 45° at all three time points failed to show any significance. Attribution could also be assigned to different amounts of MRC in the remnant. ACL injuries occur most frequently at the femoral side of the attachment, as it usually arises from impingement between femoral condyles and the ACL^25^. This means that there is a large amount of MRC in the tibial remnant stump of the injured ACL^4^. The amount of MRC in the remnant was mostly composed of Golgi organs and Ruffini receptors, which adapt slowly and mainly function through detection of static joint position^7^. MRC in the remnant were activated when mechanical deformation of the tissue took place, and started to send neural signals to the central nervous system (CNS) indicating the direction and speed of joint motion. However, the magnitude and frequency of the signal varied with the magnitude and frequency of MRC recruitment^26^. As the remnant was under greater tensile stress at the starting point than at the mid‐range of ROM, deformation was greater and more MRC were recruited. Therefore, the accuracy of JPS at the starting point of ROM was more attributed to the amount of MRC preserved, hence the amount of remnant preserved. Lee *et al*.^19^ demonstrated similar results in their study, with good results in the group with more remnant preservation at 15° and 30° for the RPP test.

The significant differences between Tegner scores for the two groups could be a manifestation of the earlier recovery of proprioceptive function. The consistency between these two aspects of postoperative evaluation could be ascribed to the positive effect of earlier recovery on faster return to daily work and sports. In addition, the psychological preparation for returning to normal life was also largely influenced by better proprioceptive function after the surgery. The complaint of “I can't feel my knee” could tremendously delay the progression of functional rehabilitation, which could negatively impact further recovery of proprioception.

The ACL's primary function is to maintain mechanical stability of the knee joint. However, several studies have failed to show a superior result with remnant preservation in terms of mechanical stability compared with standard ACLR^23^, ^27^, ^28^. The present study demonstrated a consistent conclusion that the parameters of the two groups on mechanical stability, tested by the KT2000 arthrometer at different angles and force, were equivalent. After all, even though the preservation of remnant might facilitate the ligamentization, synovialization, and vascularization of the graft, it did not necessarily increase the strength. According to Takashi's study^24^, remnant preservation did not improve the structural properties of the graft. The restoration of mechanical stability still largely depends on the tensile strength of the graft. A similar situation occurred with the postoperative Lysholm score. No significant difference could be found between the two groups. Reconstructed stability and evaluation score are not always consistent with postoperative function nor patient satisfaction^29^.

As the basic unit for proprioception, MRC have also been found in multiple knee structures other than ACL, including the posterior cruciate ligament (PCL), the meniscus, joint capsules, and skin^5^, ^10^, ^30^. A network was formed by the afferent nerves of these MRC and transmitted the impulse received to the CNS. Current studies share a common flaw of failure to exclude cases with concomitant meniscus injury^19^, ^21^, ^27^. It is impossible to assign responsibility when there are concomitant injuries alongside ACL rupture, and confounding factors might be introduced that could interfere with the credibility of the study^31^. The present study excluded the cases with injuries of other MRC containing structures to carry out a more precise allocation.

The injury‐to‐surgery time interval could also affect postoperative recovery of knee proprioception. A rapid degeneration of MRC in the remnant at 6 months post‐injury was reported by most works^9^, ^32^. In that context, the preservaton of remnant with a injury‐to‐surgery time interval longer than 6 months could bring little to none effect as the most MRCs have already degenerated and are not functional anymore. However, most patients only consider medical consultation when the symptoms are beyond manageable, which usually takes longer than 6 months. As a result, the patient population in most studies had an average injury‐to‐surgery interval longer than 6 months^8^, ^19^. The actual effect of remnant preservation would be greatly undermined under this circumstance. The present study had relatively narrow inclusion criteria, only enrolling patients injured within 6 months, excluding the confounding degenerative effect.

The contralateral limb was not used as a control in this study. Evidence has shown that deficiency of proprioception in one knee may affect the contralateral knee^30^. In addition, diminished activation in several sensorimotor cortical areas and increased activation in the pre‐supplementary motor area, the posterior secondary somatosensory area, and the posterior inferior temporal gyrus was observed using functional MRI, suggesting reorganization in the motor cortex following injury of the ACL^33^. Even though some studies suggest positive results^17^, ^34^, it is only reasonable because the task of patient's daily activity largely relies on the contralateral healthy knee after surgical management, which would no doubt make the contralateral limp stronger and more sensitive in regards to proprioceptive function.

### Limitations

Certain limitations exist in the present study. A second‐look arthroscopy would provide more data in regards to the quality and quantity of the integration between graft and remnant, and a correlation analysis between the proprioceptive function and the second‐look arthroscopic result would be able to verify the significance of the relationship. In addition, a lack of unified criteria for the measurement of proprioceptive function is a common flaw shared by every study measuring proprioceptive function. Different equipment and parameters are used in different studies, making the result less comparable.

### Conclusion

The present study demonstrated faster recovery of postoperative proprioception with more than 1/3 remnant preserved. In addition, the proprioception 15° of extension was better restored with more remnant preserved. A better Tegner score was observed for the same group. Therefore, an earlier rehabilitation targeting the mid‐range of ROM might be advantageous for the recovery of proprioceptive function.
